# Response to the Letter to the Editor Regarding “Bicarbonate and Serum Lab Markers as Predictors of Mortality in the Trauma Patient”

**DOI:** 10.5811/westjem.41519

**Published:** 2025-02-12

**Authors:** Matthew M. Talbott, Dietrich Jehle, Krishna Paul

**Affiliations:** University of Texas, Department of Emergency Medicine, Galveston, Texas

Dear Authors:

We sincerely appreciate your thoughtful comments on our article, “Bicarbonate and Serum Lab Markers as Predictors of Mortality in the Trauma Patient.” Your detailed analysis and enthusiasm regarding our study are highly valued and encourage further exploration of the topics we addressed.

We agree with the points raised regarding more narrowly stratifying the observations by trauma types and anatomical locations, as well as the nuanced approach to interpreting serum marker levels like lactate, are insightful and align with our intentions to promote more precise clinical applications of such markers.

It is appropriate to acknowledge that this database lacks the granularity necessary to differentiate between bicarbonate or lactate values obtained within the same day. This limitation inherently restricts our ability to explore the temporal progression of these markers and their association with patient outcomes, such as lactate clearance or persistence, which are indeed important prognostic factors. Your replication of our analysis with a narrower focus on anatomical injuries and lactate stratification further substantiates the value of specificity in trauma research. We are encouraged by your findings and agree that refining study cohorts could yield even more clinically relevant insights.

Thank you again for engaging with our work and for contributing to the ongoing dialogue in trauma care research. Your feedback will undoubtedly inspire future investigations aimed at improving patient outcomes.

Sincerely,

Dr. Matt Talbott, Krishna Paul, and Dr. Dietrich Jehle

Link to Letter to the Editor: https://westjem.com/articles/comments-on-bicarbonate-and-serum-lab-markers-as-predictors-of-mortality-in-the-trauma-patient.html

